# ANKHD1 is required for SMYD3 to promote tumor metastasis in hepatocellular carcinoma

**DOI:** 10.1186/s13046-018-1011-0

**Published:** 2019-01-15

**Authors:** Zhenyu Zhou, Hai Jiang, Kangsheng Tu, Wei Yu, Jianlong Zhang, Zhigang Hu, Heyun Zhang, Dake Hao, Pinbo Huang, Jie Wang, Aijun Wang, Zhiyu Xiao, Chuanchao He

**Affiliations:** 1 Guangdong Province Key Laboratory of Malignant Tumor Epigenetics and Gene Regulation, Research Center of Medicine, Sun Yat-Sen Memorial Hospital, Sun Yat-Sen University, Guangzhou, 510120 China; 2 Department of Hepatobiliary Surgery, Sun Yat-Sen Memorial Hospital, Sun Yat-Sen University, No. 33 Yingfeng Road, Guangzhou, 510289 China; 3 Department of General Surgery, The First Affiliated Hospital of Nanchang University, Nanchang, 330006 China; 4 Department of Hepatobiliary Surgery, The First Affiliated Hospital of Xi’an Jiaotong University, Xian, 710061 China; 5 Department of Hepatobiliary Surgery, The Second Affiliated Hospital of Nanchang University, Nanchang, 330006 China; 6 Surgical Bioengineering Laboratory, Department of Surgery, School of Medicine, University of California Davis, Research II, Suite 3005, 4625 2nd Avenue, Sacramento, CA 95817 USA

**Keywords:** Hepatocellular carcinoma, Metastasis, SMYD3, ANKHD1, Slug

## Abstract

**Background:**

Tumor metastasis is the major reason for poor prognosis of hepatocellular carcinoma (HCC) patients after hepatic resection. SMYD3 has been demonstrated to promote liver tumor metastasis in mice. However, the detailed molecular mechanism is still largely unknown.

**Methods:**

The effect of SMYD3 on invasiveness and metastasis of HCC was analyzed by immunohistochemistry, migration assay, invasion assay, wound healing assay and in vivo lung metastasis assay. Mass spectrometry analysis was conducted using proteins pulled down by H3K4me3 antibody in SMYD3-overexpressing cells. Luciferase reporter, chromatin immunoprecipitation, Electrophoretic mobility shift assay were used to measure the regulation of *SLUG* transcription by SMYD3-ANKHD1. In addition, the role of SMYD3-ANKHD1 in determining clinical outcomes for HCC patients was investigated by immunohistochemistry in 243 HCC tissues.

**Results:**

SMYD3 was an independent prognostic factor of HCC and promoted migration and invasion of human HCC cells. ANKHD1 was identified by mass spectrometry as a co-regulator with SMYD3. ANKHD1 interacted with H3K4me3 when cells were overexpressing SMYD3. The pro-migratory and pro-invasive effects of SMYD3 were attenuated when ANKHD1 was knocked down by siRNA. Furthermore, we found that SMYD3 bound and activated the *SLUG* gene promoter in a manner associated with elevating H3K4me3, H3K9Ac and H3K14Ac. Knockdown of ANKHD1 could attenuate the SMYD3-dependent activation of Slug expression. We further detected the expression of SMYD3 and ANKHD1 in 243 HCC patients and found that patients with positive coexpression of SMYD3 and ANKHD1 (SMYD3^+^ANKHD1^+^) had the shortest overall and recurrence-free survival.

**Conclusion:**

Our findings provide a novel molecular mechanism for the SMYD3-regulated HCC migration and metastasis, and indicates that SMYD3-ANKHD1 may be a potential target for treating HCC.

**Electronic supplementary material:**

The online version of this article (10.1186/s13046-018-1011-0) contains supplementary material, which is available to authorized users.

## Background

Although many therapeutic methods have been applied in the treatment of hepatocellular carcinoma (HCC), HCC is still ranked the second leading cause of cancer-related death globally [1]. High morbidity of metastasis is the major reason for poor prognosis of HCC patients, even of those with curative surgical treatment [2]. Thus, understanding the mechanisms of HCC metastasis will provide new opportunities for preventing HCC patients from poor treatment outcomes.

SET and MYND domain-containing protein 3 (SMYD3) is a lysine methyltransferase that was first identified and characterized in 2004 [3]. Although subsequent studies have identified SMYD3 as a H4K5 methyltransferase, H2A.Z.1 K101 dimethyltransferase, and protein lysine methyltransferase [4–6], SMYD3 plays the most critical role through its H3K4 methyltransferase activity [7, 8]. Until now, many oncogenes have been demonstrated to be regulated on transcriptional level by SMYD3 through trimethyl-H3K4 (H3K4me3) modification, which highlights the role of SMYD3 as an essential epigenetic regulator in cancer cells [9]. Furthermore, accumulated evidence suggests that SMYD3 is now considered to play a fundamental role in human tumorigenesis [8, 10–12]. These information suggest that SMYD3 may also act as an epigenetic regulator in promoting HCC development and progression. Actually, recent studies have indicated that SMYD3 is associated with liver carcinogenesis of mice and poor prognosis of HCC patients [7, 13, 14]. However, the detailed molecular mechanism of how SMYD3 promotes HCC metastasis needs further investigation.

Post-translational modifications (PTM) of histones, like H3K4me3, are recognized by so-called “reader” proteins via specialized binding modules [15]. In most cases, the binding of a reader to its cognate histone PTM can stimulate a series of molecular processes such as protein recruitment and histone modifications, and finally affect gene expression [16]. ANKHD1 (Ankyrin Repeat and KH Domain Containing 1), also known as MASK1, contains two domains: ankyrin repeats domain and KH domain [17], among which the ankyrin repeats domain has been demonstrated to be able to bind H3K9me2, H3K9me1 [18]. In recent years, ANKHD1 has been shown as a potential biomarker in various cancers [19–21]. However, whether ANKHD1 could bind a specific histone mark and serve as an tumor promoter in HCC is not yet known.

In this study, by conducting mass spectrometry analysis, we identified ANKHD1 as a co-regulator with SMYD3. We provided evidence that SMYD3 transactivates its target genes and promotes HCC cells migration and invasion through ANKHD1. Moreover, we found that SMYD3-ANKHD1 correlates with the prognosis of HCC patients.

## Methods

### Patients and follow-up

A total of 243 patients with HCC who underwent curative resection between 2010 and 2013 at the Sun Yat-Sen Memorial Hospital were enrolled in this study. This study was conducted in accordance with the Declaration of Helsinki. The study protocol was approved by the research ethics committee of Sun Yat-Sen Memorial Hospital, and written informed consent was obtained from each patient. The diagnosis of HCC was based on pathology and dynamic contrast-enhanced imaging (computed tomography scan or magnetic resonance imaging). Briefly, the inclusion criteria were: (1) distinctive pathologic diagnosis of HCC, (2) no anti-cancer treatment before hepatic resection, (3) suitable formalin-fixed, paraffin-embedded tissues, and (4) complete clinicopathologic data. The detailed clinical characteristics of the 243 patients are summarized in Supplementary Table S1. The TNM classification for HCC was based on The American Joint Committee on Cancer/TNM Staging for Liver Tumors (7th edition, 2010). The median follow-up of this cohort was 39.34 months.

In addition, a total of 25 pairs of primary HCC tissues and major portal vein tumor thrombi (mPVTTs) were collected after surgical resection for further immunohistochemistry. The study protocol was approved by the research ethics committee of Sun Yat-Sen University. Written informed consents were obtained from these patients.

### In vivo lung metastasis assay

All animal experiments were performed in accordance with current Chinese regulations and standards regarding the use of laboratory animals, and approved by the animal ethics committee of Sun Yat-sen University. In brief, cells were injected into ten 4-week-old BALB/c nude mice intravenously (1 × 10^6^ cells/ mouse). OMe-modified control and ANKHD1 siRNA were intravenously injected into the mice from tail vein (5 μg/g/mouse) twice a week at the second week after tumor cells injection. After 4 weeks, the mice were euthanized and sacrificed. Lungs of the mice were removed and fixed in Bouin’s solution for 24 h. The number of lung surface metastatic foci was then counted. Lungs were excised and embedded in paraffin for further study.

### Subcellular fractionation

Cytoplasmic and nuclear fractions were prepared from 5 × 10^7^ cells by using Minute™ Cytoplasmic & Nuclear Extraction Kits (Invent Biotechnologies, USA). Briefly, cells were washed with ice-cold PBS and lysed for 30 min on ice with cytoplasmic lysis buffer. The lysates were centrifuged at 14000 rpm for 5 min at 4 °C, and the supernatants (cytoplasmic fractionation) were collected in a fresh tube kept on ice. The pellet was resuspended with nuclear lysis buffer. The homogenate was incubated on ice for 30 min on ice and centrifuged at 14000 rpm for 5 min at 4 °C. Supernatant was collected as the nuclear fraction. The cytoplasmic and nuclear fractions were then analyzed using western blot.

### Co-immunopreciptation (co-IP)

Total protein lysate was obtained in immunoprecipitation buffer. 500 μg of total protein was mixed with 1 μg the primary antibody, or IgG, and the mixture were shaken on a rotating shaker at 4 °C for 1 h. Beads (Santa, USA) were added to the mixture and shaken at 4 °C overnight. Then the beads were collected by centrifugation at 2500 rpm for 5 min at 4 °C, and then washed 4 times by immunoprecipitation buffer. 5× sample loading buffer was added to the beads before boiling for 5 min. The supernatant was collected and detected using western blot.

### Luciferase reporter assay

The Slug luciferase reporter plasmid (pGL4.18-Slug-WT) was generated by inserting a DNA fragment which contained the snai2 5′-flanking region from − 359 to + 109 into pGL4.18 vector (Promega, USA). Mutation reporter plasmids (pGL4.18-Slug-M1 and pGL4.18-Slug-M2) were made by replacing the two SMYD3 binding sequences (5’-CCCTCC-3′ to 5’-CAAGAC-3′) in pGL4.18-Slug-WT using the MutanBEST Kit (TaKaRa, Japan) according to the manufacturer’s instructions. Primer sequences of plasmid construction are listed in Additional file 1: Table S7.

Cells were transfected with pGL4.18-Slug-WT, pGL4.18-Slug-M1 or pGL4.18-Slug-M2 combined with pCDNA3.1-SMYD3, siANKHD1 or negative control using ViaFect™ Transfection Reagent (Promega, USA). For each set of transfections, pGL4.74 (Promega, USA) was always included to control transfection efficiency. At 48 h after transfection, luciferase activity was measured using a dual luciferase reporter assay system (Promega, USA) according to the manufacturer’s instructions.

### Electrophoretic mobility shift assay (EMSA)

Biotin end-labeled probe was prepared by Sangon Biotech (China). EMSA was performed using the LightShift® Chemiluminescent EMSA Kit (Pierce, USA) according to the manufacturer’s instructions. DNA binding reactions were performed in 20 μL system containing biotin-labeled oligonucleotides and nuclear extracts. Additional unlabeled oligonucleotides were added for competition. Reaction products were then separated by electrophoresis. Thereafter, the protein-DNA complexes were transferred onto a positively charged nylon membrane (Millipore, USA) and detected by chemiluminescence.

### Chromatin immunoprecipitation assay (ChIP)

ChIP assays were performed using EZ-CHIP Kit according to the manufacturer’s instruction (Millipore, USA). Briefly, cells were cross-linked using 1% formaldehyde. After washing with PBS, the cells were resuspended in 1 mL of SDS lysis buffer containing protease inhibitor cocktail. DNA was sheared to small fragments (200–300 bp) by sonication. The sheared chromatin was immunoprecipitated with different antibodies (Additional file 2) overnight at 4 °C. DNA from immunoprecipitation and the input samples was analyzed using SYBR Green qPCR Master Mix (Biotool, USA). Primers were shown in Additional file 1: Table S7.

### Statistical analysis

Statistical analysis was performed with SPSS software (version 17.0). Cumulative survival and recurrence rates were calculated by the Kaplan-Meier analysis and the log-rank test. Based on the variables selected on univariate analysis, the multivariate Cox proportional hazards model was used to determine the independent prognostic factors of HCC. Quantitative data and categorical data were analyzed by the Student’s *t* test and Fisher’s exact test, respectively. *P* < 0.05 was considered statistically significant.

## Results

### SMYD3 promotes HCC cells migration and invasion

**Fig. 1:**
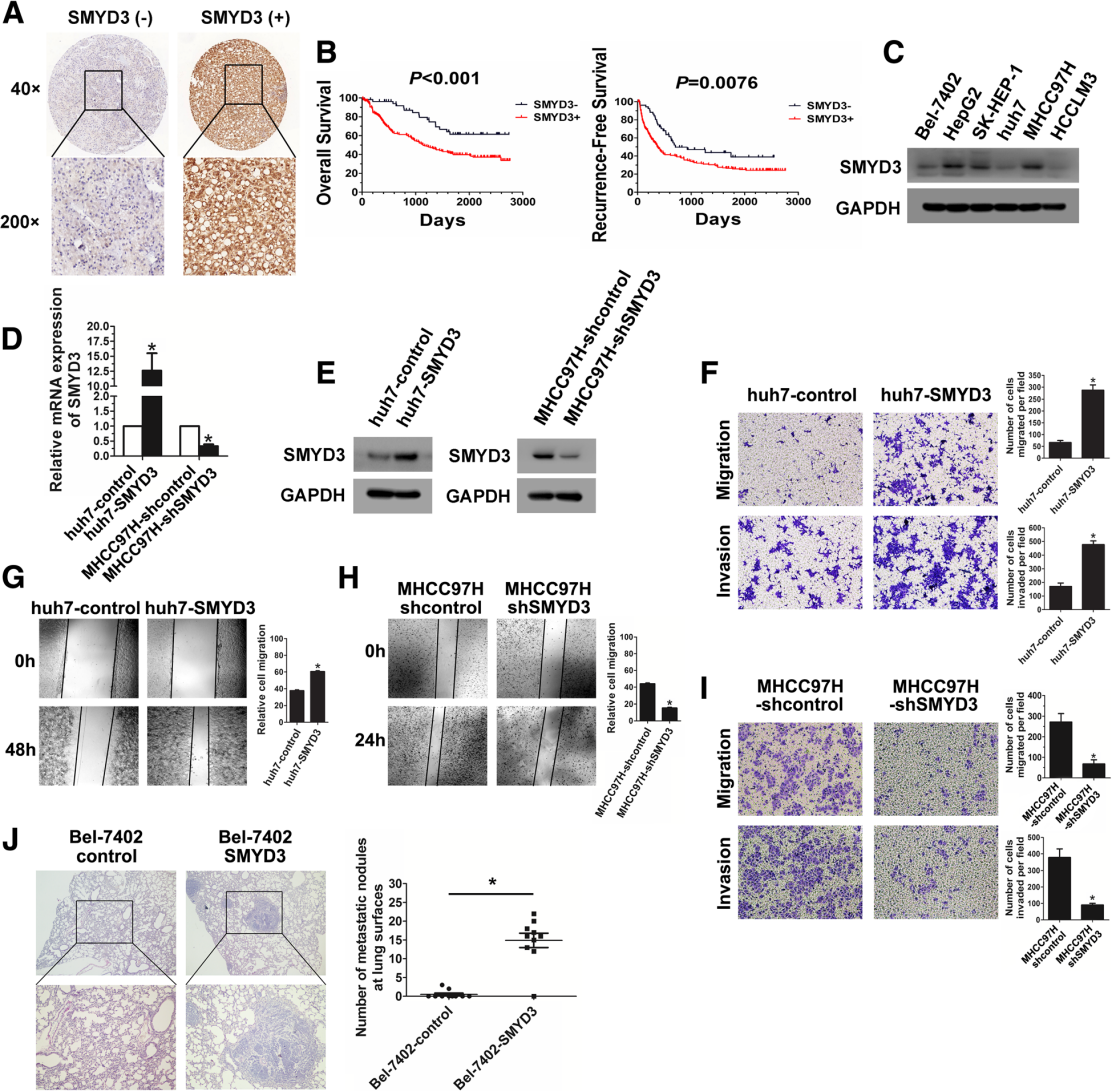
SMYD3 is associated with poor prognosis of HCC and promotes HCC cells migration and invasion. **a** IHC analysis of SMYD3 expression in HCC tissues. **b** Kaplan-Meier analysis of the overall survival or recurrence-free survival in HCC patients according to SMYD3 expression. **c** Western blot analysis of SMYD3 expression in different HCC cell lines. **d)** and **e** Real-time PCR and western blot confirmed the efficiencies of SMYD3 stable overexpression in huh7 cells and stable knockdown in MHCC97H cells, respectively. **f** and **g** Wound healing assay, migration assay and invasion assay were performed in SMYD3-overexpressing cells. **h** and **i** Wound healing assay, migration assay and invasion assay were performed in SMYD3 knockdown cells. **j** In vivo lung metastasis assay was used to evaluate the in vivo effects of SMYD3 on tumor metastasis by tail vein injections of cells; representative images of H&E-stained sections were derived from lung metastatic nodules of both groups; Number of metastatic lung foci observed at the surface of the lungs of mice was counted in each group (*n* = 6). Data are presented as mean ± SD for three independent experiments. *P < 0.05

We first assessed SMYD3 expression in a tissue microarray of 243 HCC samples. SMYD3 expression was found in 188 of 243 (77.4%) primary HCC tissues (Fig. 1a). SMYD3 positive expression in HCC was significantly associated with HBV infection, microvascular invasion, poor tumor differentiation, and high TNM stage (Additional file 1: Table S1). Furthermore, HCC patients with positive SMYD3 expression had shorter overall and recurrence-free survival compared with those with negative SMYD3 expression (Fig. 1b). Cox’s multivariate proportional hazards model indicated that the expression of SMYD3 was an independent predictor of survival (*P* = 0.012) and recurrence (*P* = 0.028) in HCC patients after curative resection (Additional file 1: Table S2).

To further explore the oncogenic mechanism of SMYD3 in HCC, we chose Bel-7402 and huh7 to establish cell lines that stably overexpressed SMYD3 (Fig. 1c; Additional file 3: Figure. S1A). Besides, MHCC97H was chosen to establish SMYD3 stable knockdown cell line (Fig. 1c; Additional file 3: Figure S1A). Both upregulation and knockdown of SMYD3 expression were confirmed by real-time PCR and western blot (Fig. 1d and e; Additional file 3:Figure S1B- S1D). We found that upregulation of SMYD3 significantly enhanced the migration and invasion capacities of huh7 and Bel-7402 cells (Fig. 1f and g; Additional file 3: Figure S1E and S1F). In contrast, knockdown of SMYD3 markedly reduced cell migration and invasion in MHCC97H cells (Fig. 1h and i). To evaluate the in vivo effects of SMYD3 on HCC metastasis, Bel-7402-SMYD3 cells were injected into the tail veins of nude mice. The incidence of lung metastasis in the SMYD3-overexpressing group was significantly higher than that in the control group (Fig. 1j). These results suggest that SMYD3 is an important factor in during HCC progression.

### Identification of ANKHD1 as a co-regulator with SMYD3

**Fig. 2:**
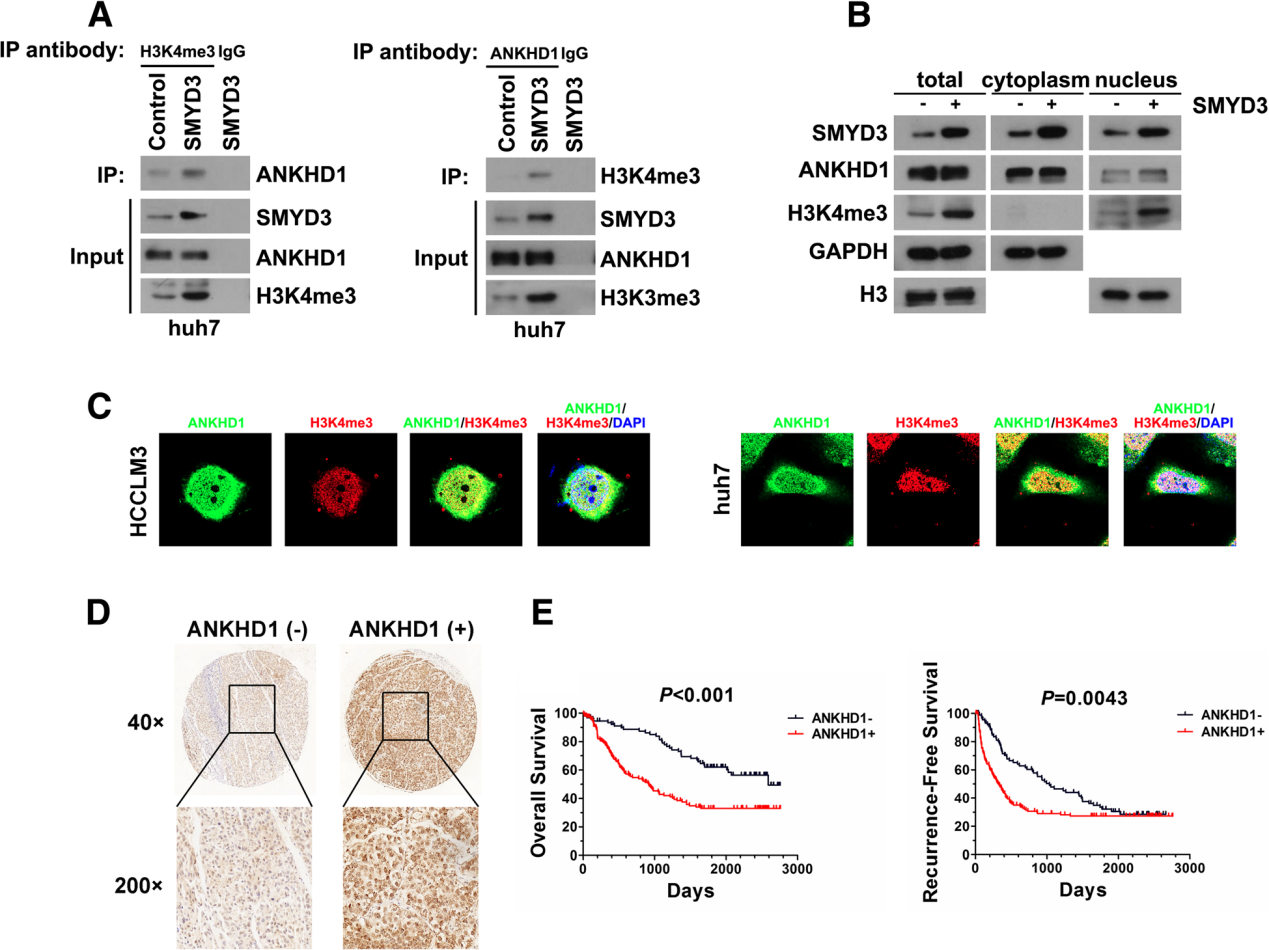
ANKHD1 is a co-regulator with SMYD3 and is associated with poor prognosis of HCC. **a** Lysates of huh7-SMYD3 and huh7-control cells were immunoprecipitated for endogenous H3K4me3 or ANKHD1 and immunoblotted for ANKHD1 or H3K4me3, respectively. **b** Subcellular fractions were isolated to analyze the coexpression of SMYD3, ANKHD1 and H3K4me3 in cytoplasm and nucleus using western blot. **c** Confocal analysis of HCCLM3 and huh7 cells transfected with SMYD3 displaying ANKHD1 (green), H3K4me3 (red) and DAPI (blue) staining; MERGE shows the overlapped images. **d** IHC analysis of SMYD3 expression in HCC tissues. **e** Kaplan-Meier analysis of the overall survival or recurrence-free survival in HCC patients according to ANKHD1 expression

SMYD3 plays the most critical role through its H3K4 methyltransferase activity [8]. To investigate whether there exist proteins that bind to H3K4me3 under SMYD3 overexpression and influence target genes expression, we applied mass spectrometry analysis using proteins pulled down by H3K4me3 antibody in SMYD3-overexpressing cells. Interestingly, we found ANKHD1 could specifically bind to H3K4me3 when cells were overexpressing SMYD3 (Additional file 1: Table S3). Our further validation by Co-IP showed that SMYD3 overexpression lead to the interaction between ANKHD1 and H3K4me3, whereas knockdown of SMYD3 inhibited these interactions (Fig. 2a; Additional file 3: Figure S2A, S2C and S2D). Moreover, subcellular fractionation and immunofluorescent staining showed that ANKHD1 and H3K4me3 co-localized in the nuclei when SMYD3 was overexpressed (Fig. 2b and c; Additional file 3: Figure S2B). In addition, we also detected the co-localization of SMYD3 and ANKHD1. Results showed that SMYD3 was colocalized with ANKHD1 in both cytoplasm and nucleus, especially in cytoplasm (Additional file 3: Figure S2E). These data suggest that ANKHD1 may be involved in regulating the expression of SMYD3-targeted genes.

### ANKHD1 expression correlates with metastatic potential and patients survival of HCC

ANKHD1 is overexpressed in multiple malignant tumors, such as acute leukemias [19], renal cancer cell [20], and breast cancer [21]. However, little is known in HCC. Our IHC results in tissue microarray showed that 150 of 243 (61.7%) primary HCC tissues have positive ANKHD1 expression (Fig. 2c). Positive expression of ANKHD1 was significantly associated with large tumor size, microvascular invasion, multiple nodules, poor tumor differentiation, and high TNM stage (Additional file 1: Table S1). HCC patients with positive ANKHD1 expression were associated with significantly shorter overall and recurrence-free survival (Fig. 2d). Moreover, the expression of ANKHD1 in HCC cells was higher as the invasion potential of HCC cells increased. (Additional file 3: Figure S2F). ANKHD1 overexpression significantly enhanced the migration and invasion capacities of HCC cell line, Bel-7402 (Additional file 3: Figure S2G-S2I). These data suggest that, similar to SMYD3, ANKHD1 is also associated with the aggressiveness of HCC progression.

### ANKHD1 mediates the pro-migratory and pro-invasive effects of SMYD3

**Fig. 3:**
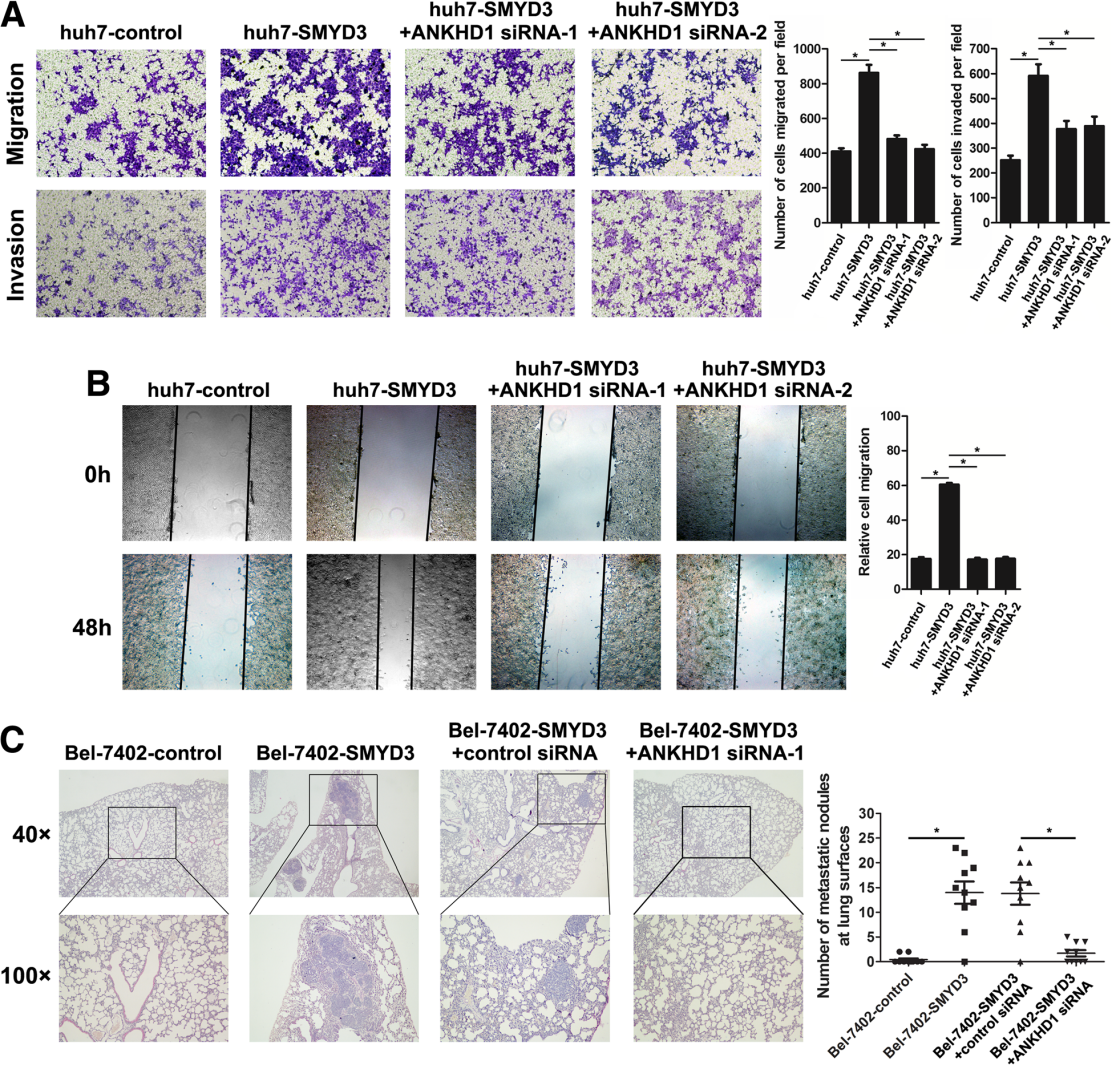
ANKHD1 mediates the pro-migratory and pro-invasive effects of SMYD3. **a** Migration and invasion assay in stable huh7-SMYD3 cells with ANKHD1 knockdown or not. **b** Wound healing assay of huh7-SMYD3 cells with ANKHD1 knockdown or not. **c** In vivo lung metastasis assay was performed using Bel-7402 control, Bel-7402-SMYD3, and Bel-7402-SMYD3 combined with ANKHD1 siRNA or control siRNA injection; representative images of H&E-stained sections were derived from lung metastatic nodules of the four experimental groups; Number of metastatic lung foci observed at the surface of the lungs of mice was counted in each group (*n* = 6 for each group). Data are presented as mean ± SD for three independent experiments. *P < 0.05

To investigate whether ANKHD1 could regulate oncogenic mechanism of SMYD3 in HCC, huh7-SMYD3 cells were transfected with siRNA to knock down ANKHD1 expression. ANKHD1 knockdown significantly reduced SMYD3-enhanced cell migration and invasion (Fig. 3a and b). Moreover, inhibition of ANKHD1 expression in SMYD3-overexpressing cells significantly attenuated the incidence of lung metastasis induced by SMYD3 in vivo (Fig. 3c). Interestingly, overexpressing ANKHD1 in the condition of SMYD3 knockdown did not influence the migration and nvasion capacities of MHCC97H (Additional file 3: Figure S3A and S3B). These data suggests that SMYD3 promotes HCC invasion and metastasis through ANKHD1.

### Identification Slug as a target of SMYD3-ANKHD1 in HCC

**Fig. 4:**
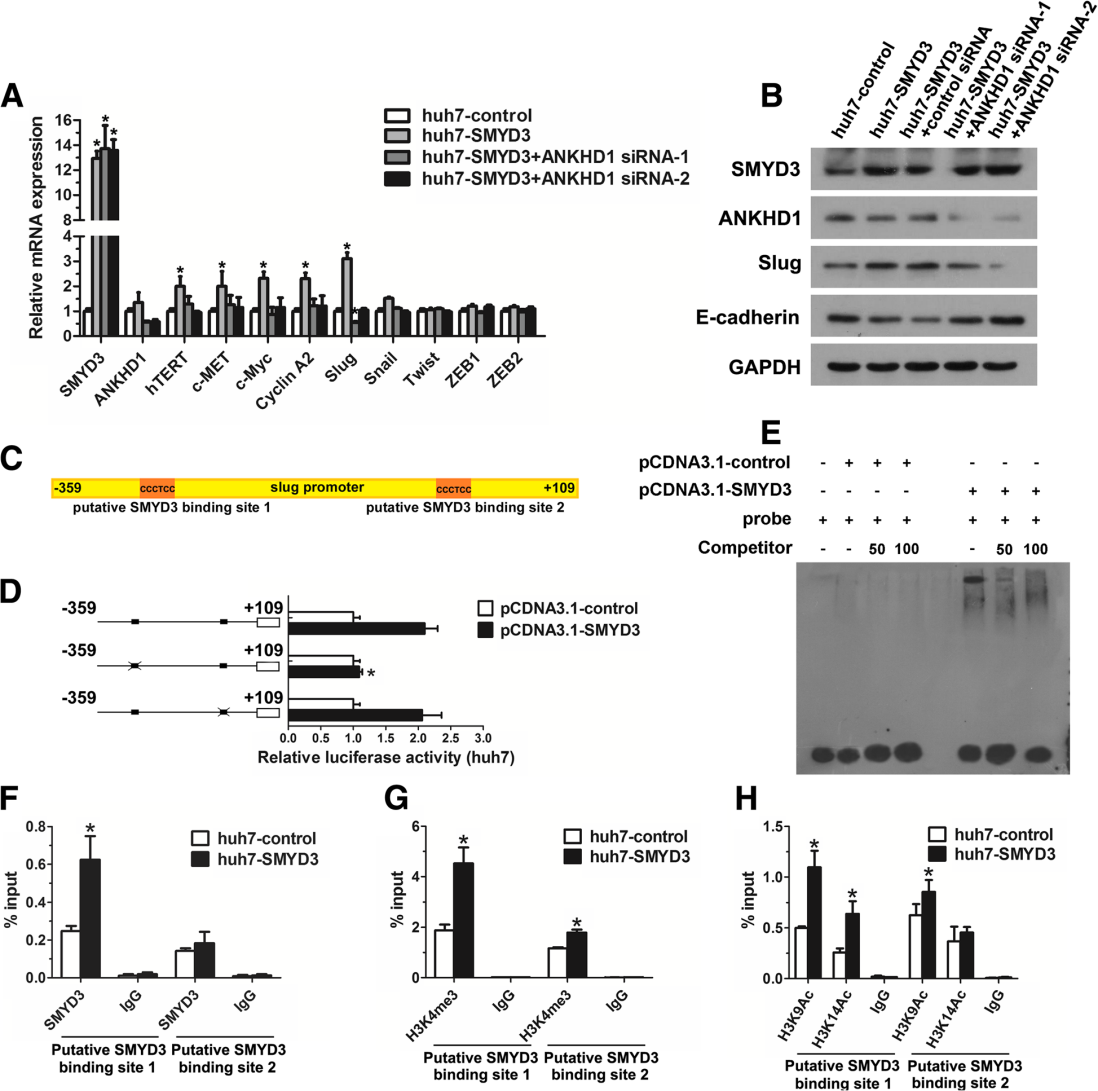
Identification Slug as a target of SMYD3-ANKHD1 in HCC. **a** Real-time PCR was performed to detect the mRNA expression of SMYD3-dependent target genes and EMT- inducing transcription factors in huh7 cells stably overexpressing SMYD3 with or without ANKHD1 knockdown. **b** Western blot detection of the expression of E-cadherin and Slug in huh7 cells stably overexpressing SMYD3 with or without ANKHD1 knockdown. **c** Two putative SMYD3 binding sites in human *SLUG* gene promoter region. **d** Luciferase reporter assay of huh7 cells cotransfected with the indicated luciferase reporter (wide type *SLUG* gene promoter-luciferase construct or its SMYD3 binding site mutants) and the empty vector or SMYD3 expression vector. **e** Nuclear extracts prepared from cells treated with pCDNA3.1-SMYD3 or pCDNA3.1-control were incubated with the biotin-labeled oligonucleotides probe corresponding to binding site 1 in the *SLUG* gene promoter to perform EMSA. Different fold excess of unlabeled oligonucleotides probe for binding site 1 were used to compete with the interaction between the labeled probe and SMYD3. **f-h** ChIP assays were performed in huh7-control and huh7-SMYD3 stable cell lines using antibodies against SMYD3, H3K4me3, and H3K9, K14Ac; immunoprecipitated DNA was measured by real-time PCR using primers for amplifying the SMYD3-binding regions in the *SLUG* gene promoter. Data are shown as mean ± SD of three separate experiments. *P < 0.05

Given the findings above, we sought to determine whether SMYD3-ANKHD1 regulated SMYD3 target genes in HCC cells. We found that ANKHD1 could mediate the upregulation of SMYD3 target genes (Fig. 4a). In addition, because the results above showed that SMYD3-ANKHD1 promoted HCC cells migration and invasion, we also detected the expression of five EMT-inducing transcription factors. Our results showed that the mRNA expression of Slug was regulated by SMYD3-ANKHD1, in contrast, no differences were found in the other four transcription factors (Fig. 4a). Results from western blot in SMYD3 stable overexpressing or knockdown cells and cells with SMYD3 inhibition, and IHC in 25 pairs of primary HCC tissues, mPVTTs, as well as microvascular invasion (MVIs) further supported the regulation of Slug by SMYD3 (Additional file 3: Figure S4A-S4C, Figure S5A; Additional file 1: Table S4). Furthermore, knockdown of ANKHD1 significantly reduced SMYD3-dependent expression of Slug, and attenuated the loss of E-cadherin expression on protein levels (Fig. 4b). We therefore selected Slug for the following investigation.

SMYD3 is known to bind to a putative motif CCCTCC in its target genes promoters [22]. Interestingly, we identified two putative SMYD3 binding elements within the *SLUG* gene promoter region (Fig. 4c). To determine whether SMYD3 regulated Slug transcription and define which putative binding site was responsible for the regulation, luciferase reporter assay was performed. Results showed that SMYD3 enhanced *SLUG* gene promoter activity, but the enhancement was abolished when the binding site 1 was mutated (Fig. 4d), indicating that SMYD3 activates Slug transcription through binding site 1. Then, we performed EMSA and ChIP assay to determine whether SMYD3 physically bound to the *SLUG* gene promoter. EMSA showed that oligonucleotides probe corresponding to binding site 1 of the *SLUG* gene promoter strongly combined with SMYD3, and these SMYD3-labeled probe complexes were abrogated by the unlabeled oligonucleotides (Fig. 4e). We next performed ChIP assay to determine whether the interaction exists under physiological conditions. As shown in Fig. 4F, increasing binding of SMYD3 to binding site 1 of the *SLUG* gene promoter was found in cells overexpressing SMYD3. Furthermore, SMYD3 upregulation increased H3K4 trimethylation, H3K9 and H3K14 acetylation in the same region, while SMYD3 inhibition by BCI-121 decreased H3K4 trimethylation, indicating that SMYD3 is required for increasing trimethylation of H3K4 and acetylation of H3K9/14 in the *SLUG* gene promoter (Fig. 4g and h; Additional file 3: Figure S5B and S5C). Taken together, these results suggest that SMYD3 is an epigenetic regulator of Slug expression in HCC, which results in H3K4 trimethylation and subsequent H3K9, K14 acetylation in the *SLUG* gene promoter.

### SMYD3 transactivates Slug expression through ANKHD1

**Fig. 5:**
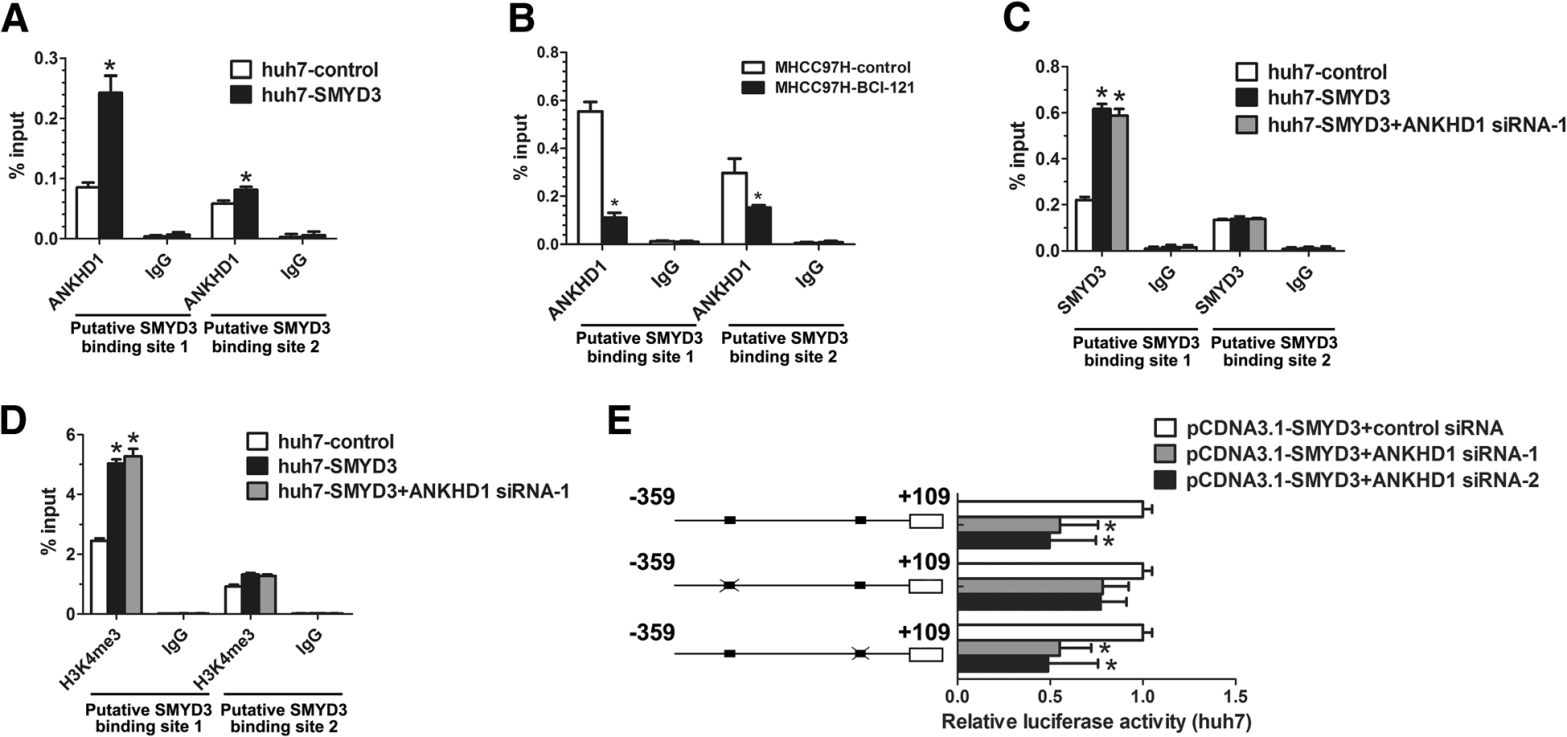
SMYD3 transactivates Slug expression through ANKHD1. **a** ChIP assays were performed in huh7-control and huh7-SMYD3 stable cell lines using antibodies against ANKHD1; immunoprecipitated DNA was measured by real-time PCR using primers for amplifying the SMYD3-binding regions in the *SLUG* gene promoter. **b** ChIP assays were performed in MHCC97H with and without SMYD3 inhibitor (BCI-121) treatment using antibodies against ANKHD1; immunoprecipitated DNA was measured by real-time PCR using primers for amplifying the SMYD3-binding regions in the SLUG gene promoter. c and **d** ChIP assays were performed in huh7-control and huh7-SMYD3 stable cell lines with or without ANKHD1 knockdown using antibodies against SMYD3 and H3K4me3; immunoprecipitated DNA was measured by real-time PCR using primers for amplifying the SMYD3-binding regions in the SLUG gene promoter. (E) Luciferase reporter assay of huh7 cells cotransfected with the indicated luciferase reporter (wide type SLUG gene promoter-luciferase construct or its SMYD3 binding site mutants), the empty vector or SMYD3 expression vector, and control siRNA or ANKHD1 siRNA. Data are shown as mean ± SD of three separate experiments. *P < 0.05

As our above results showed that ANKHD1 bind to H3K4me3 under SMYD3 overexpression, we next investigated whether ANKHD1 mediates the transactivation of *SLUG* gene by SMYD3. We found that SMYD3 overexpression recruited significantly more ANKHD1 to binding site 1 of the *SLUG* gene promoter than binding site 2, while SMYD3 inhibition by BCI-121 prevented this recruitment (Fig. 5a and b). However, knockdown of ANKHD1 did not affect the recruitment of SMYD3 or the trimethylation of H3K4 on the *SLUG* gene promoter (Fig. 5c and d). Interestingly, the attenuated effect of ANKHD1 knockdown on *SLUG* gene promoter activity in SMYD3-overexpressing cells was significantly weakened when the binding site 1 was mutated (Fig. 5e). Together, these data suggest that SMYD3-dependent increase of H3K4 trimethylation can recruit ANKHD1 to *SLUG* gene promoter, which mediates the SMYD3-induced transactivation of *SLUG* gene promoter.

### SMYD3-ANKHD1 correlate with HCC patient outcomes

**Fig. 6:**
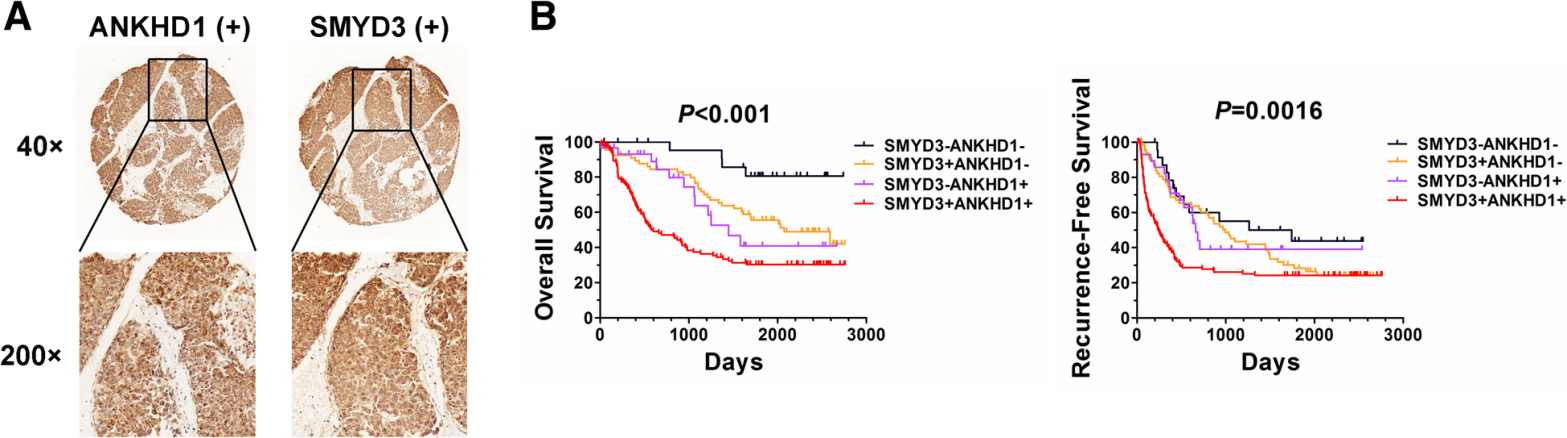
SMYD3-ANKHD1 correlate with HCC patient outcomes. **a** HCC tissue with concurrent SMYD3 and ANKDH1 positive expression. **b** Kaplan-Meier analysis of the overall survival or recurrence-free survival in HCC patients according to the concurrent expression of SMYD3 and ANKHD1

To explore the role of SMYD3-ANKHD1 in determining clinical outcomes for HCC patients, we assessed the correlation between SMYD3-ANKHD1 and the prognosis of 243 HCC patients. Although no significant relationship was found between the expression level of SMYD3 and ANKHD1 in the 243 HCC tissues (Fig. 6a; Additional file 3: Figure S6A-S6C; Additional file 1: Table S5), Kaplan-Meier analysis revealed that patients with positive coexpression of SMYD3 and ANKHD1 (SMYD3^+^ANKHD1^+^) had the shortest overall and recurrence-free survival (Fig. 6b). Noteworthily, there were no significant differences in recurrence-free survival rates among patients with SMYD3^+^ANKHD1^−^, SMYD3^−^ANKHD1^+^ or SMYD3^−^ANKHD1^−^ (Fig. 6b; Additional file 3: Figure S6D-S6F). Furthermore, we also assessed the correlation between Slug and SMYD3/ ANKHD1 expression in 243 HCC clinical samples. We found that Slug expression was correlated with ANKHD1 expression in SMYD3 positive HCC patients, but not in SMYD3 negative HCC patients, suggesting both SMYD3 and ANKHD1 are necessary for Slug expression in HCC (Additional file 3: Figure S7; Additional file 1: Table S6). These data provide clinical evidence that ANKHD1 acts as a co-regulator with SMYD3 in promoting HCC progression.

## Discussion

Many histone modifications have been implicated in influencing gene expression. For instance, the methylation of H3K4, H3K36, H3K79 and the acetylation of H3K9, H3K14 are closely correlated with transcriptional activation; whereas the methylation of H3K9, H3K27 and H4K20 is associated with gene repression [23]. Deregulation of these histone modifications will lead to changes in the expression of several oncogenes that ultimately result in cancer development and progression [24]. Thus, it is critical to understand the underlying epigenetic molecular mechanisms in HCC. SMYD3, as a histone H3K4 methyltransferase, has received considerable attention in the last few years due to its critical roles in multiple malignant processes, especially in tumor invasion and metastasis [8, 22]. Recent study has shown that SMYD3 was associated with EMT in HCC [7]. However, the exact role of SMYD3 in promoting tumor invasion and metastasis in HCC remains to be elucidated. In this study, we demonstrated that SMYD3 was frequently upregulated in human HCC tissues and was positively correlated with microvascular invasion, malignant differentiation, and advanced TNM stage. Multivariate analysis revealed that SMYD3 expression level was an independent and significant risk factor for survival and recurrence of HCC after curative resection. Further studies showed that SMYD3 overexpression promoted HCC cells migration and invasion via transactivating Slug expression. This transactivation was through increasing trimethylation of H3K4 and acetylation of H3K9/14 in the *SLUG* gene promoter. Our data indicate that SMYD3 acts as an epigenetic regulator in HCC, resulting in HCC cells invasion and metastasis.

There are three histone lysine methylation states, including monomethylation, dimethylation and trimethylation (me1, me2 and me3, respectively). Unlike histone lysine acetylation, which decreases the positive charge thus weakens electrostatic interactions between histones and DNA, and reduces chromatin compaction, histone lysine methylation cannot change the electronic charge of the amino-acid side chain [24]. Therefore, histone lysine methylation should function through effectors which specifically recognize the methylated site and regulate the transcriptional activity [25]. These “reader” proteins generally contain methyl-lysine-binding motifs, such as PHD, chromo, tudor, PWWP, WD40, BAH, ADD, ankyrin repeat, MBT and zn-CW domains [26].

The methylated lysine-binding characteristic of ankyrin repeat domain was first reported in 2008, as a functional domain of G9a and GLP [18]. Robert and his colleagues showed that G9a and GLP could not only methylate H3K9, but also bind H3K9me1 and H3K9me2 through their ankyrin repeat domain, leading to transcriptional repression [18]. In this study, we reported another ankyrin repeat domain containing protein, ANKHD1, could interact with H3K4me3 when cells were overexpressing SMYD3. Moreover, our further analysis showed that ANKHD1 could bind to the specific region at *SLUG* gene promoter in a SMYD3-dependent manner and mediate the SMYD3-induced transactivation of *SLUG* gene promoter in HCC cell lines. In fact, previous studies have shown ANKHD1 was an oncogenic protein that could activate target genes transcription [21, 27]. In addition, our data also showed that ANKHD1 expression was associated with patient outcomes in HCC and could mediate the pro-migratory and pro-invasive effect of SMYD3, which was not reported before. Therefore, our data reveal a new mechanism for ANKHD1 in promoting tumor progression, serving as a “reader” protein coordinating with SMYD3 to promote target gene expression. However, further study is needed to clarify the exact binding mechanism between ANKHD1 and H3K4me3.

A major hallmark of aggressive HCC is its metastatic capacity that can be characterized as vascular invasion or intrahepatic metastasis [28]. Portal vein invasion is the most common event among HCC patients with vascular invasion [29]. Our results showed that SMYD3 expression was positively correlated with Slug expression, but inversely correlated with E-cadherin expression in not only HCC primary tissues, but also their vascular invasion tissues (MVIs and mPVTTs). These findings strongly suggest that SMYD3/Slug pathway plays an important role in promoting HCC metastasis and may be a useful biomarker for HCC metastasis and poor prognosis.

## Conclusions

In summary, our study suggests that SMYD3-ANKHD1 axis is a new molecular mechanism for the regulation of the target genes of SMYD3, such as Slug, which promotes the invasion and metastasis of HCC. Targeting SMYD3-ANKHD1 signaling may provide a potential therapeutic opportunities against advanced HCC.

## Additional Files

Additional File 1: **Table S1.** Association between SMYD3, ANKHD1 expression and patient's clinicopathologic features in HCCs. **Table S2.** Univerate and multivariate analysis of factors associated with survival and recurrence of 243 HCCs. **Table S3.** Mass spectrometry analysis of the proteins interacted with H3K4me3 when SMYD3 was overexpressed. **Table S4**. Association between SMYD3 and Slug expression in HCC, MVI and mPVTT. **Table S5.** Association between SMYD3 and ANKHD1 expression in 243 HCCs. **Table S6.** Association between the expression of ANKHD1 and Slug in SMYD3 positive/ negative HCC. **Table S7.** siRNA/shRNA sequences used in the study. **Table S8.** Primer sequences used in the study. (DOCX 43 kb)

Additional File 2: Supplementary Methods and materials. (DOCX 20 kb)

Additional File 3: Supplemental figure legends. (DOCX 8542 kb)

## Abbreviations

ANKHD1: Ankyrin Repeat and KH Domain Containing 1
EMT: Epithelial-mesenchymal transition
H3K4me3: Histone 3 lysine 4 trimethylation
mPVTT: Major portal vein tumor thrombi
MVI: Microvascular invasion
SMYD3: SET and MYND domain-containing protein 3
