# Supplementary material for: ANKHD1 is required for SMYD3 to promote tumor metastasis in hepatocellular carcinoma
Source: J Exp Clin Cancer Res. 2019 Jan 15;38:18. doi: 10.1186/s13046-018-1011-0 (PMC6332640; doi:10.1186/s13046-018-1011-0)
Supplement: Supplementary file 1 — Table S1. Association between SMYD3, ANKHD1 expression and patient's clinicopathologic features in HCCs. Table S2. Univerate and multivariate analysis of factors associated with survival and recurrence of 243 HCCs. Table S3. Mass spectrometry analysis of the proteins interacted with H3K4me3 when SMYD3 was overexpressed. Table S4. Association between SMYD3 and Slug expression in HCC, MVI and mPVTT. Table S5. Association between SMYD3 and ANKHD1 expression in 243 HCCs. Table S6. Association between the expression of ANKHD1 and Slug in SMYD3 positive/ negative HCC. Table S7. siRNA/shRNA sequences used in the study. Table S8. Primer sequences used in the study. (DOCX 43 kb) [file 13046_2018_1011_MOESM1_ESM.docx]

**Table S1. Association between SMYD3, ANKHD1 expression and patient's clinicopathologic features in HCCs**

| Clinicopathological Variables | | Case Number  (n=243) | SMYD3 expression | | *P* Value | ANKHD1 expression | | *P* Value |
| --- | --- | --- | --- | --- | --- | --- | --- | --- |
|  |  |  | negative（n=55） | positive（n=188） |  | negative（n=93） | positive（n=150） |  |
| Age |  |  | 51.27±11.5 | 50.8±12.3 | 0.80 | 52.05±11.9 | 50.19±12.2 | 0.244 |
| Sex | Female | 21 | 7 | 14 | 0.273 | 8 | 13 | 0.986 |
|  | male | 222 | 48 | 174 |  | 85 | 137 |  |
| Serum AFP | ≤400 ng/mL | 148 | 37 | 111 | 0.346 | 64 | 84 | 0.058 |
|  | >400 ng/mL | 95 | 18 | 77 |  | 29 | 66 |  |
| HBsAg | Negative | 24 | 17 | 7 | <0.001* | 10 | 14 | 0.825 |
|  | Positive | 219 | 38 | 181 |  | 83 | 136 |  |
| Cirrhosis | Absent | 70 | 18 | 52 | 0.500 | 23 | 47 | 0.309 |
|  | Present | 173 | 37 | 136 |  | 70 | 103 |  |
| Tumor size | ≤5 cm | 104 | 29 | 75 | 0.121 | 65 | 39 | <0.001* |
|  | >5 cm | 139 | 26 | 113 |  | 28 | 111 |  |
| Microvascular invasion | Absent | 92 | 29 | 63 | 0.012* | 65 | 27 | <0.001* |
|  | Present | 151 | 26 | 125 |  | 28 | 123 |  |
| Number of tumors | Single | 180 | 41 | 139 | 0.928 | 77 | 103 | 0.016* |
|  | Multiple | 63 | 14 | 49 |  | 16 | 47 |  |
| Tumor differentiation | I-II | 142 | 44 | 98 | <0.001* | 70 | 72 | <0.001* |
|  | III-IV | 101 | 11 | 90 |  | 23 | 78 |  |
| TNM stage | I-II | 141 | 39 | 102 | 0.030* | 73 | 68 | <0.001* |
|  | III-IV | 102 | 16 | 86 |  | 20 | 82 |  |

Abbreviations: AFP, α-fetoprotein; HBsAg, hepatitis B surface antigen; TNM, tumor-lymph node-metastasis. * *P*<0.05

**Table S2. Univerate and multivariate analysis of factors associated with survival and recurrence of 243 HCCs**

| **Variables** | **Survival** | | | | | | **Recurrence** | | | | | |
| --- | --- | --- | --- | --- | --- | --- | --- | --- | --- | --- | --- | --- |
|  | **Univariate Analysis** | | | **Multivariate Analysis** | | | **Univariate Analysis** | | | **Multivariate Analysis** | | |
|  | HR | 95% CI | *P* value | HR | 95% CI | P value | HR | 95% CI | P value | HR | 95% CI | *P* value |
| **Age**  (≤50.0 vs. >50.0) | 0.933 | 0.653-1.334 | 0.704 |  |  |  | 0.784 | 0.575-1.069 | 0.124 |  |  |  |
| **Sex**  (female vs. male) | 1.048 | 0.548-2.002 | 0.888 |  |  |  | 1.121 | 0.658-1.908 | 0.675 |  |  |  |
| **Serum AFP**  (≤400 vs. >400ng/mL) | 1.653 | 1.154-2.366 | 0.006* |  |  |  | 1.531 | 1.117-2.098 | 0.008* |  |  |  |
| **HBsAg**  (negative vs. positive) | 0.933 | 0.514-1.694 | 0.819 |  |  |  | 0.991 | 0.582-1.686 | 0.972 |  |  |  |
| **Cirrhosis**  (absent vs. present) | 1.141 | 0.767-1.696 | 0.516 |  |  |  | 1.322 | 0.929-1.882 | 0.121 |  |  |  |
| **Tumor size**  (≤5 vs. >5cm) | 2.585 | 1.757-3.804 | <0.001* |  |  |  | 2.030 | 1.469-2.807 | <0.001* |  |  |  |
| **Microvascular invasion**  (absent vs. present) | 2.207 | 1.491-3.267 | <0.001* |  |  |  | 1.727 | 1.244-2.396 | 0.001* |  |  |  |
| **Number of tumors**  (single vs. multiple) | 1.568 | 1.061-2.318 | 0.024* |  |  |  | 1.511 | 1.077-2.120 | 0.017* |  |  |  |
| **Tumor differentiation**  (I-II vs. III-IV) | 1.940 | 1.355-2.776 | <0.001* |  |  |  | 1.550 | 1.134-2.118 | 0.006* |  |  |  |
| **TNM stage**  (I-II vs. III-IV) | 3.581 | 2.484-5.162 | <0.001* | 2.481 | 1.584-3.887 | <0.001* | 3.216 | 2.339-4.422 | <0.001* | 2.597 | 1.748-3.858 | <0.001* |
| **SMYD3 expression**  **(** negative vs. positive) | 2.343 | 1.384-3.966 | 0.002* | 1.987 | 1.159-3.405 | 0.012* | 1.745 | 1.153-2.643 | 0.009* | 1.617 | 1.054-2.480 | 0.028* |

Abbreviations: AFP, α-fetoprotein; HBsAg, hepatitis B surface antigen; TNM, tumor-lymph node-metastasis. * *P*<0.05

**Table S3. Mass spectrometry analysis of the proteins interacted with H3K4me3 when SMYD3 was overexpressed**

| Number | Protein name | Full name |
| --- | --- | --- |
| 1 | ANKHD1 | Ankyrin repeat and KH domain containing 1 |
| 2 | TRIP13 | Thyroid hormone receptor interactor 13 |
| 3 | RNF168 | Ring finger protein 168 |
| 4 | NLRC4 | NLR family CARD domain containing 4 |
| 5 | CDKL4 | Cyclin dependent kinase like 4 |
| 6 | NCOA5 | Nuclear receptor coactivator 5 |
| 9 | INO80 | INO80 complex subunit |
| 10 | Vimentin | Vimentin |
| 11 | OTX2 | Orthodenticle homeobox 2 |
| 12 | FCF1 | rRNA-processing protein |
| 14 | POLB | DNA polymerase beta |
| 15 | RRM1 | Ribonucleotide reductase catalytic subunit M1 |
| 16 | YTHDC1 | YTH domain containing 1 |
| 17 | MRE11A | MRE11 homolog, double strand break repair nuclease |

|  |
| --- |

|  | |  | SMYD3 | | *P* value |
| --- | --- | --- | --- | --- | --- |
|  |  |  | Negative | Positive |  |
| Slug | HCC | Negative | 7 | 6 | 0.030 |
|  |  | Positive | 1 | 11 |  |
|  | MVI and mPVTT | Negative | 6 | 4 | 0.007 |
|  |  | Positive | 1 | 14 |  |
| E-cadherin | HCC | Negative  Positive | 2  6 | 14  3 | 0.010 |
|  | MVI and mPVTT | Negative  Positive | 3  4 | 17  1 | 0.012 |

**Table S4. Association between SMYD3 and Slug expression in HCC, MVI and mPVTT**

|  | | SMYD3 | | *P* value |
| --- | --- | --- | --- | --- |
|  |  | Negative | Positive |  |
| ANKHD1 | Negative | 26  29 | 67  121 | 0.155 |
|  | Positive |  |  |  |

**Table S5. Association between SMYD3 and ANKHD1 expression in 243 HCCs**

**Table S6. Association between the expression of ANKHD1 and Slug in SMYD3 positive/ negative HCC.**

|  | **SMYD3 positive** | ANKHD1 | | *P* value |
| --- | --- | --- | --- | --- |
|  |  | Negative (n=67) | Positive (n=121) |  |
| Slug | Negative (n=88) | 42 | 46 | 0.001* |
|  | Positive (n=100) | 25 | 75 |  |
|  | **SMYD3 negative** | ANKHD1 | | *P* value |
|  |  | Negative (n=26) | Positive (n=29) |  |
| Slug | Negative (n=43) | 21 | 22 | 0.659 |
|  | Positive (n=12) | 5 | 7 |  |

* *P*<0.05

**Table S7. siRNA/shRNA sequences used in the study.**

| Name | | sequences |
| --- | --- | --- |
| **siRNA:** | |  |
| siSMYD3-1 | sense: | 5'-UCACAGCUGUGACCCCAACTT-3' |
|  | santisense: | 5'-GUUGGGGUCACAGCUGUGATT-3' |
| siSMYD3-2 | sense: | 5'-AGCCUGAUUGAAGAUUUGATT-3' |
|  | antisense: | 5'-UCAAAUCUUCAAUCAGGCUTT-3' |
| siANKDH1-1 | sense: | 5’-GGAGAAAGCCUGCUGUGUUTT-3’ |
|  | antisense: | 5’-AACACAGCAGGCUUUCUCCTT-3’ |
| siANKDH1-2 | sense: | 5’-GCAGGCAUCAACACUCAUUTT-3’ |
|  | antisense: | 5’-AAUGAGUGUUGAUGCCUGCTT-3’ |
| **shRNA:** |  |  |
| shSMYD3 | sense: | 5’-CCGGAGCCTGATTGAAGATTTGATTCTC  GAGAATCAAATCTTCAATCAGGCTTTTTTG-3’ |
|  | antisense: | 5’-AATTCAAAAAAGCCTGATTGAAGATTTG  ATTCTCGAGAATCAAATCTTCAATCAGGCT-3’ |

**Table S8. Primer sequences used in the study.**

| Primer name | | | Primer sequences |
| --- | --- | --- | --- |
| **Primers for real-time PCR:** | | |  |
|  | | SMYD3 sense: | 5'-GTCTTCAAACTTATGGATGGAGC-3' |
|  | | SMYD3 antisense: | 5'-GGCATCCTGTATTTCTTCTCTCA-3' |
|  | | ANKHD1 sense | 5’-CACTGTCCTCACGAGTTGCT-3’ |
|  | | ANKHD1 antisense | 5’-AGAAACTCGCTGGGAAGGTG-3’ |
|  | | Slug sense: | 5'-TGCTGCCAAATCATTTCAACTG-3' |
|  | | Slug antisense: | 5'-CAACCAGACAACCGACATGT-3' |
|  | | Snail sense: | 5'-GTAATGGCTGTCACTTGTCG-3' |
|  | | Snail antisense: | 5'-TGTAAACATCTTCCTCCCAGG-3' |
|  | | ZEB1 sense: | 5'-GGCATACACCTACTCAACTACGG-3' |
|  | | ZEB1 antisense: | 5'- TGGGCGGTGTAGAATCAGAGTC-3' |
|  | | ZEB2 sense: | 5'- GCTGGACACGATTGCACATT-3' |
|  | | ZEB2 antisense: | 5'- AGAACTAAGCGTGTGGGAAG-3' |
|  | | Twist sense: | 5'- GCCGGAGACCTAGATGTCATTG-3' |
|  | | Twist antisense: | 5'- CACGCCCTGTTTCTTTGAATTT-3' |
|  | | hTERT sense: | 5'- TGTCAAGGTGGATGTGACGG-3' |
|  | | hTERT antisense: | 5'- CATGTACGGCTGGAGGTCTG-3' |
|  | | C-met sense: | 5'- CTGGTGCCACGACAAATGTG-3' |
|  | | C-met antisense: | 5'- GTCAGCCTTGTCCCTCCTTC-3' |
|  | | C-myc sense: | 5'- CATCAGCACAACTACGCAGC-3' |
|  | | C-myc antisense: | 5'- CGTTGTGTGTTCGCCTCTTG-3' |
|  | | Cyclin A2 sense: | 5'- TGAGCATGTCACCGTTCCTC-3' |
|  | | Cyclin A2 antisense: | 5'- CAGCTGGCTTCTTCTGAGCT-3' |
|  | | ANKHD1 sense | 5’-CACTGTCCTCACGAGTTGCT-3’ |
|  | | ANKHD1 antisense | 5’-AGAAACTCGCTGGGAAGGTG-3’ |
|  | | GAPDH sense: | 5’-GAAGGTGAAGGTCGGAGTCAACG-3’ |
|  | | GAPDH antisense: | 5’-TGCCATGGGTGGAATCATATTGG-3’ |
| **Primers for Slug promoter mutation construction:** | | |  |
|  | binding site 1 mutation sense: | | 5’-GCTCCTGCGCCaagaCTAGCTCCCAG-3’ |
|  | binding site 1 mutation antisense: | | 5’-CGGGGTCTCTGCCCTGCCCGCC-3’ |
|  | binding site 2 mutation sense: | | 5’-GAAAAAAAAACaagaCCAGCCAAAAC-3’ |
|  | binding site 2 mutation antisense: | | 5’-CTCTCTTTTGCAAGAAAGATCCAATC-3’ |
| **Primers used for ChIP in the Slug promoter:** | | |  |
|  | SMYD3 binding site 1 sense: | | 5’-CCTCTCCAGATGCCACTTCC-3’ |
|  | SMYD3 binding site 1 antisense: | | 5’-GATCCACGCTCTCTGGGA-3’ |
|  | SMYD3 binding site 2 sense: | | 5’-TCAGCTGTGATTGGATCTTTCT-3’ |
|  | SMYD3 binding site 2 antisense: | | 5’-GTCCCTACAGCATCGCGG-3’ |
