# Supplementary material for: ANKHD1 is required for SMYD3 to promote tumor metastasis in hepatocellular carcinoma
Source: J Exp Clin Cancer Res. 2019 Jan 15;38:18. doi: 10.1186/s13046-018-1011-0 (PMC6332640; doi:10.1186/s13046-018-1011-0)
Supplement: Supplementary file 2 — Supplementary Methods and materials. (DOCX 20 kb) [file 13046_2018_1011_MOESM2_ESM.docx]

**Supplementary Methods and materials**

**Cell lines and antibodies**

The human HCC cell lines (Bel-7402, HepG2, huh7, SK-HEP-1, PLC/PRF/5, and MHCC97H) and HEK293T cell line were purchased from the Cell Bank of Type Culture Collection of the Chinese Academy of Sciences, Shanghai Institute of Cell Biology, Chinese Academy of Sciences in 2014. Cells were immediately expanded and frozen such that they could be revived every 2 to 3 months. All the cells were maintained in DMEM (Gibco, USA) supplemented with 10% fetal bovine serum ( Gibco, USA). All cells were used for experiments at least 3 passages after thawing.

Antibodies used in this study are: SMYD3 (ab187149, abcam, USA), ANKHD1 (ab117788, and ab199164, abcam, USA), E-cadherin (ab40772, abcam, USA), E-cadherin (3195, Cell Signaling Technology, USA), Slug (9585, Cell Signaling Technology, USA), H3K4me3 (9727, Cell Signaling Technology, USA), H3K9Ac (06-942, Millipore, USA), H3K14ac (07-353, Millipore, USA), and GAPDH (sc-25778, Santa Cruz Biotechnology, USA).

**Immunohistochemistry**

Primary antibodies used for immunochemical staining were SMYD3 (1:200), ANKHD1 (1:200), E-cadherin (1:400), and Slug (1:100). The staining results were semi-quantitatively represented by an immunohistochemical score combined with the intensity and extent of staining. The intensity of staining was scored as 0 (negative), 1 (weak), 2 (strong). The extent of staining was scored as 0 (negative), 1 (1%-25%), 2 (26%-50%), 3 (51%-75%), and 4 (76%-100%). The total score was calculated by multiplying the staining intensity and the staining extent. Each case was finally considered negative if the total score was 0 to 1 and positive if the total score was over 2. Immunohistochemical analysis was done by two pathologists by consensus without knowledge of the clinicopathological information.

**Establishment of stable SMYD3-overexpressing cells and SMYD3** **knockdown cells**

Huh7 and Bel-7402 cell lines were used to construct SMYD3-overexpressing cells, MHCC97H cell line was used to construct SMYD3 knockdown cell. HEK293T packaging cells were transfected with the appropriate retroviral construct. Culture supernatants were collected at 48 hours after transfection, and infected target cells. Cells were then selected with puromycin for 2 weeks. ShRNAs sequences for SMYD3 are listed in Additional file 2: Table S7.

**Western blot**

In brief, proteins from lysed cells were separated by SDS-PAGE and transferred to polyvinylidene difluoride membranes (Millipore, USA). The membrane was blocked and incubated overnight at 4°C with specific antibody against SMYD3 (1:5000), GAPDH (1:2000), Slug (1:1000), E-cadherin (1:10000), ANKHD1 (1:1000), respectively. The membranes were then incubated with an HRP-conjugated secondary antibody.

**RNA interference assay**

RNA oligonucleotides prepared by GenePharma (China) were transfected into HCC cells using lipofectamine RNAiMax (Invitrogen, USA) according to the manufacturer’s instructions. The siRNA sequences used are listed in Additional file 2: Table S7.

**RNA isolation and real-time PCR assay**

Total RNA was extracted with TRIzol reagent (TaKaRa, Japan). Reverse transcription was performed with PrimeScript RT reagent Kit (TaKaRa, Japan) according to the manufacturer’s instructions. For real-time PCR analysis, the resultant cDNA products were amplified using SYBR Green qPCR Master Mix (Biotool, USA) in triplicates. Primer sequences of the genes analyzed are listed in Additional file 2: Table S8.

**Migration, invasion assay and wound healing assay**

Cells were seeded into Boyden chambers (8-µm pore size, Corning, USA) with or without Matrigel (BD Biosciences, USA) for migration or invasion assay. After 24h or 48h incubation, membranes were fixed with 4% paraformaldehyde and then stained with crysal violet. The mean number of cells was calculated by microscope from 9 consecutive fields.

For the wound healing assay, cells were grown to full confluence, maintained in serum-free DMEM, and scratched with 200 μL pipette. Migration photos were captured using a microscope at 0h, 24h, and 48h after scratching. We measured the fraction of cell coverage compared to initiate gap and defined it as the migration rate.

**Immunofluorescent (IF) Staining**

These experiments were performed as described previously [1]. Briefly, cells transfected with Flag-SMYD3 were grown in 35 mm culture dishes (NEST Biotechnology, China) to 20-40% confluence, washed three times with PBS, fixed in 4% paraformaldehyde for 40 minutes at 4°C. The cells were incubated with the first primary antibody (ANKHD1, 1:100) overnight at 4°C, followed by incubation with fluorescence-conjugated secondary antibody at room temperature for 1h. Another primary antibodies (Flag, 1:500; H3K4me3, 1:100) were then ncubated into the cells overnight at 4°C, followed by incubation with fluorescence-conjugated secondary antibody at room temperature for 1h. The samples were co-stained with 4’,6-diamidino-2-phenylindole (DAPI) and examined by a laser confocal microscope (Zeiss, German).

[1] Gu P, Chen X, Xie R, Han J, Xie W, Wang B, Dong W, Chen C, Yang M, Jiang J, er al. LncRNA HOXD-AS1 Regulates Proliferation and Chemo-resistance of Castration-resistant Prostate Cancer via Recruiting WDR5. Mol The 2017;25(8): 1959–1973.
