# Supplementary material for: ANKHD1 is required for SMYD3 to promote tumor metastasis in hepatocellular carcinoma
Source: J Exp Clin Cancer Res. 2019 Jan 15;38:18. doi: 10.1186/s13046-018-1011-0 (PMC6332640; doi:10.1186/s13046-018-1011-0)

**Supplemental figure legends**

**Figure S1. SMYD3 promotes Bel-7402** **cells migration and invasion. (A) and (B)** Statistical analyses results of figure 1c and 1e. **(C) and (D)** Real-time PCR (C) and western blot (D) confirmed the efficiencies of SMYD3 stable overexpression in Bel-7402 cells. **(E) and (F)** Wound healing assay (E), migration assay and invasion assay (F) were performed in Bel-7402-SMYD3 and its control cells. Data are presented as mean ± SD for three independent experiments. **P*<0.05

**Figure S2. ANKHD1 is a co-regulator with SMYD3 and promotes HCC cells migration and invasion.** **(A) and (B)** Statistical analyses results of figure 2a and 2b. **(C)** Lysates of Bel-7402-SMYD3 and Bel-7402-control cells were immunoprecipitated for endogenous H3K4me3 or ANKHD1 and immunoblotted for ANKHD1 or H3K4me3, respectively. **(D)** Lysates of MHCC97H-shSMYD3 and MHCC97H-shcontrol cells were immunoprecipitated for endogenous H3K4me3 or ANKHD1 and immunoblotted for ANKHD1 or H3K4me3, respectively. **(E)** Confocal analysis of HCCLM3 and huh7 cells transfected with SMYD3 displaying ANKHD1 (green), SMYD3 (Flag) (red) and DAPI (blue) staining; MERGE shows the overlapped images. **(F)** The expression of ANKHD1 was analyzed by western blot in 6 HCC cell lines. **(G)** Western blot confirmed the efficiencies of ANKHD1 overexpression in Bel-7402 cells. **(H) and (I)** Wound healing assay (I), migration assay and invasion assay (H) were performed in Bel-7402-SMYD3 and its control cells. Data are presented as mean ± SD for three independent experiments. **P*<0.05.

**Figure S3. ANKHD1 promotes HCC cells migration and invasion in a manner dependent on SMYD3. (A)** Migration and invasion assay in stable MHCC97H-shSMYD3 cells with ANKHD1 overexpression or not. **(B)** Wound healing assay of MHCC97H-shSMYD3 cells with ANKHD1 knockdown or not.

**Figure S4. SMYD3 regulates Slug expression in HCC.** **(A)** Statistical analyses results of figure 4b. **(B)** Western blot in SMYD3 stable overexpressing or knockdown cells showed Slug was positively regulated by SMYD3. **(C)** IHC analysis of SMYD3, Slug, and E-cadherin expression in primary HCC tissues, mPVTTs and MVIs.

**Figure S5. SMYD3 inhibition by BCI-121 deceases the trimethylation of H3K4 on the SLUG gene promoter**. **(A)** Western blot showed that BCI-121 treatment significantly attenuated the expression of Slug and H3K4me3 compared with control cells. **(B)** and **(C**) ChIP assays were performed in MHCC97H with and without SMYD3 inhibitor (BCI-121) treatment using antibodies against SMYD3 and H3K4me3; immunoprecipitated DNA was measured by real-time PCR using primers for amplifying the SMYD3-binding regions in the SLUG gene promoter.

**Figure S6. SMYD3-ANKHD1 correlate with HCC patient outcomes. (A)** HCC tissue with concurrent SMYD3 and ANKDH1 negative expression. **(B)** HCC tissue with SMYD3 negative and ANKDH1 positive expression. **(C)** HCC tissue with SMYD3 positive and ANKDH1 negative expression. **(D)** Kaplan-Meier analysis of the recurrence-free survival in HCC patients between SMYD3^+^ANKHD1^-^ and SMYD3^-^ANKHD1^+^ group. **(E)** Kaplan-Meier analysis of the recurrence-free survival in HCC patients between SMYD3^-^ANKHD1^-^ and SMYD3^+^ANKHD1^-^ group. **(F)** Kaplan-Meier analysis of the recurrence-free survival in HCC patients between SMYD3^-^ANKHD1^-^ and SMYD3^-^ANKHD1^+^ group.

**Figure S7.** The correlation between Slug and SMYD3/ ANKHD1 expression in HCC clinical samples. **(A)** HCC cases with SMYD3^+^ANKDH1^+^Slug^+^, SMYD3^+^ANKDH1^+^Slug^-^, SMYD3^-^ANKDH1^-^Slug^+^ and SMYD3^-^ANKDH1^-^Slug^-^. **(B)** Kaplan-Meier analysis of the overall survival and recurrence-free survival in Slug-positive HCC patients according to the concurrent expression of SMYD3 and ANKHD1.

**Figure S1**


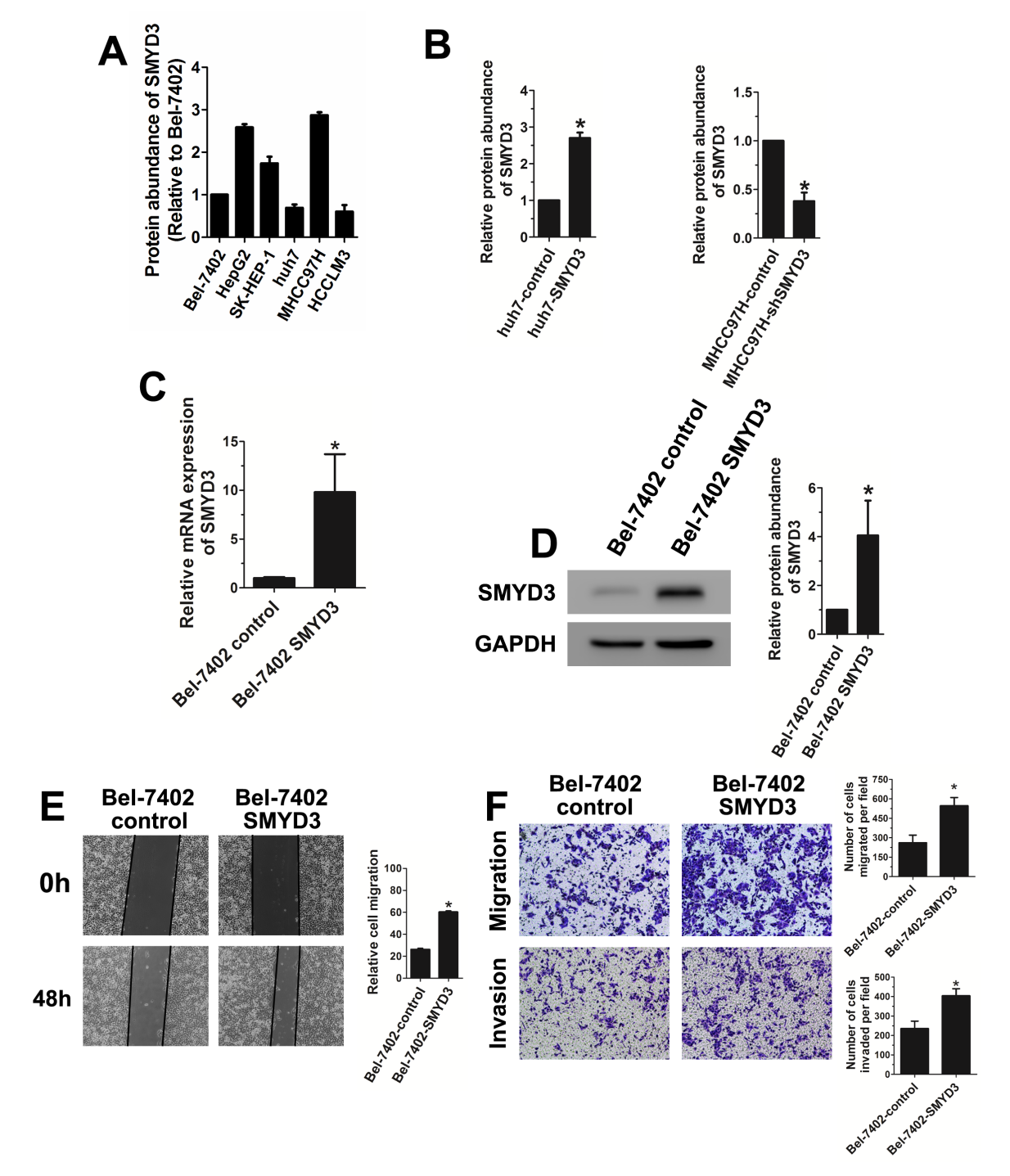


**Figure S2**


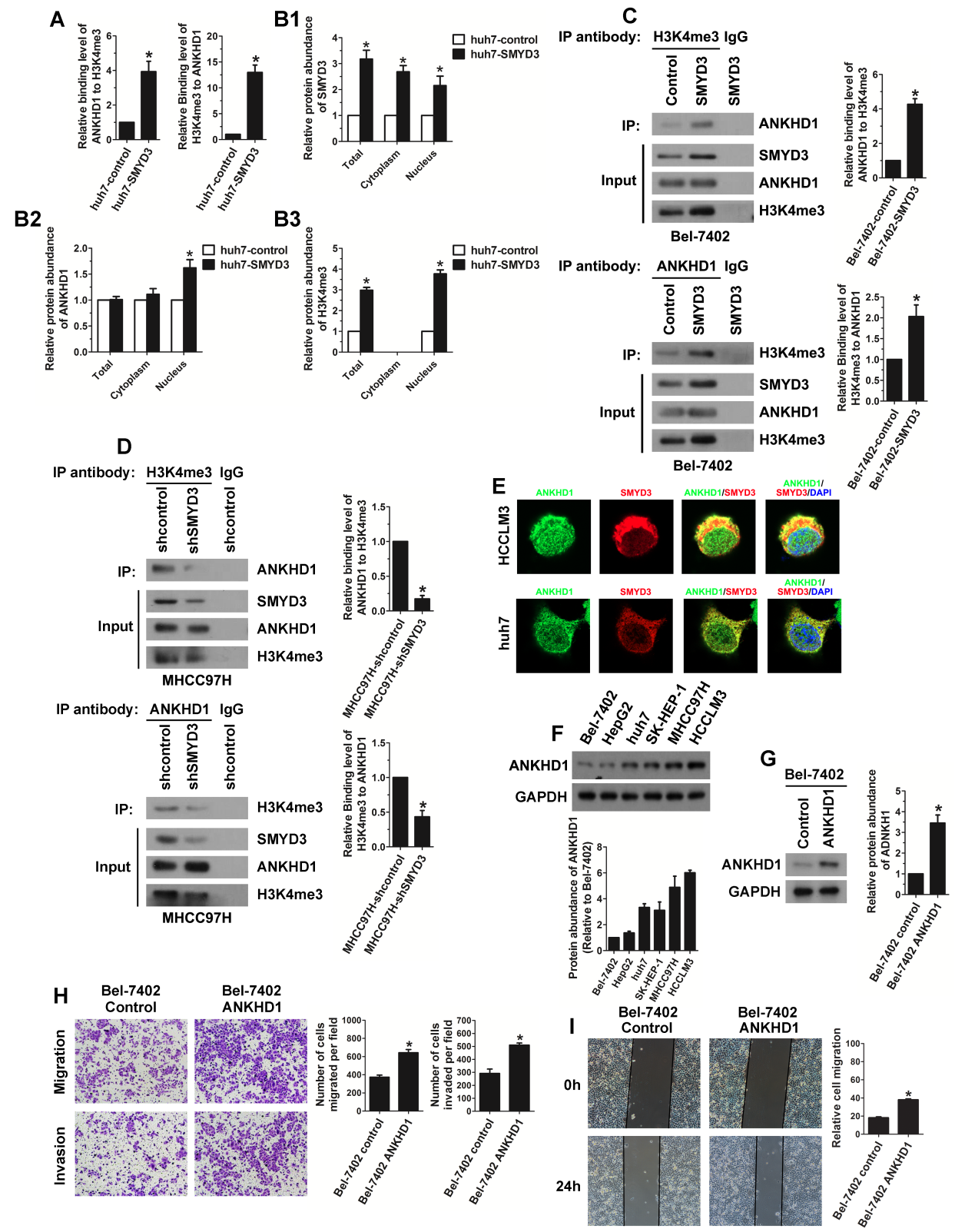


**Figure S3**


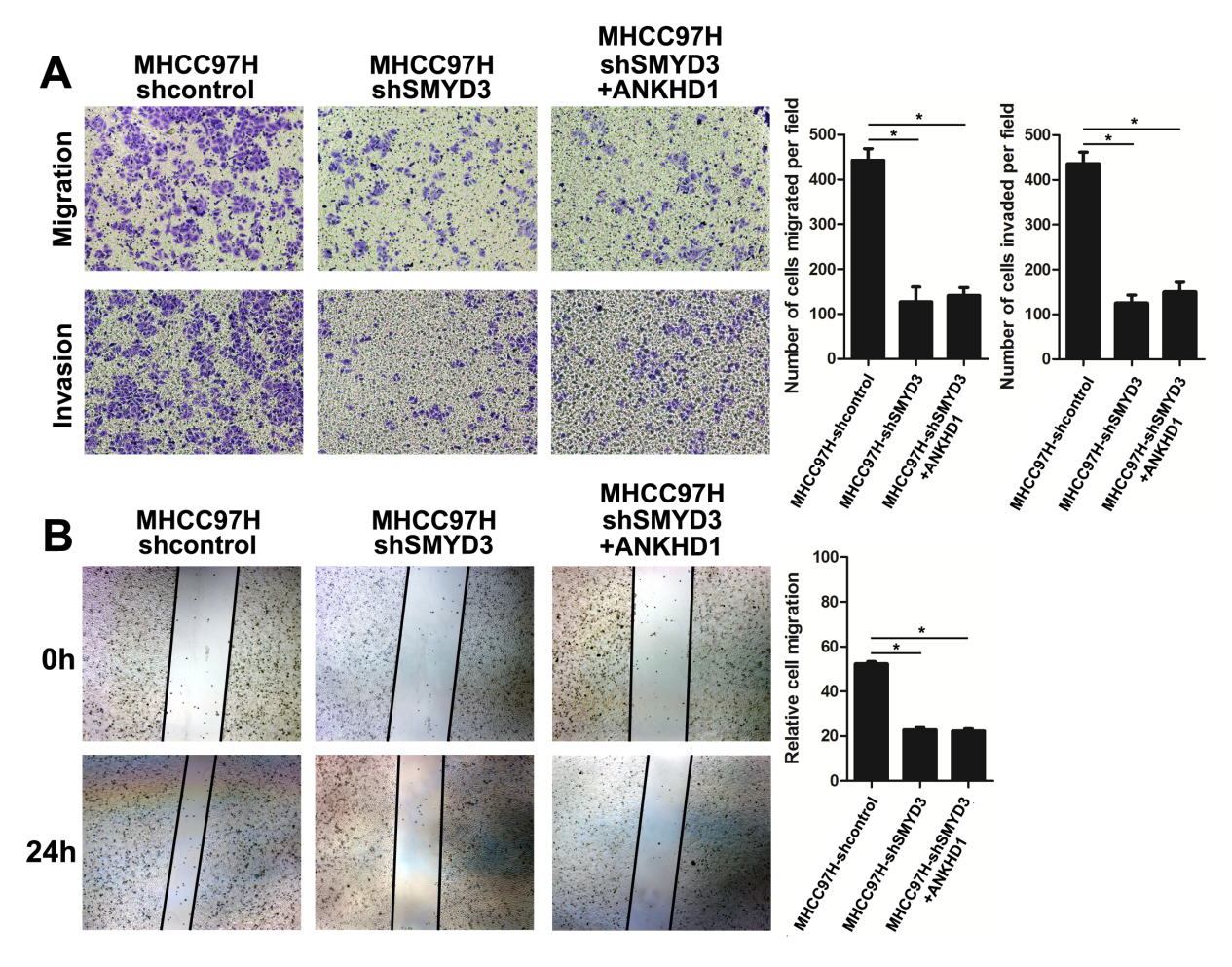


**Figure S4**


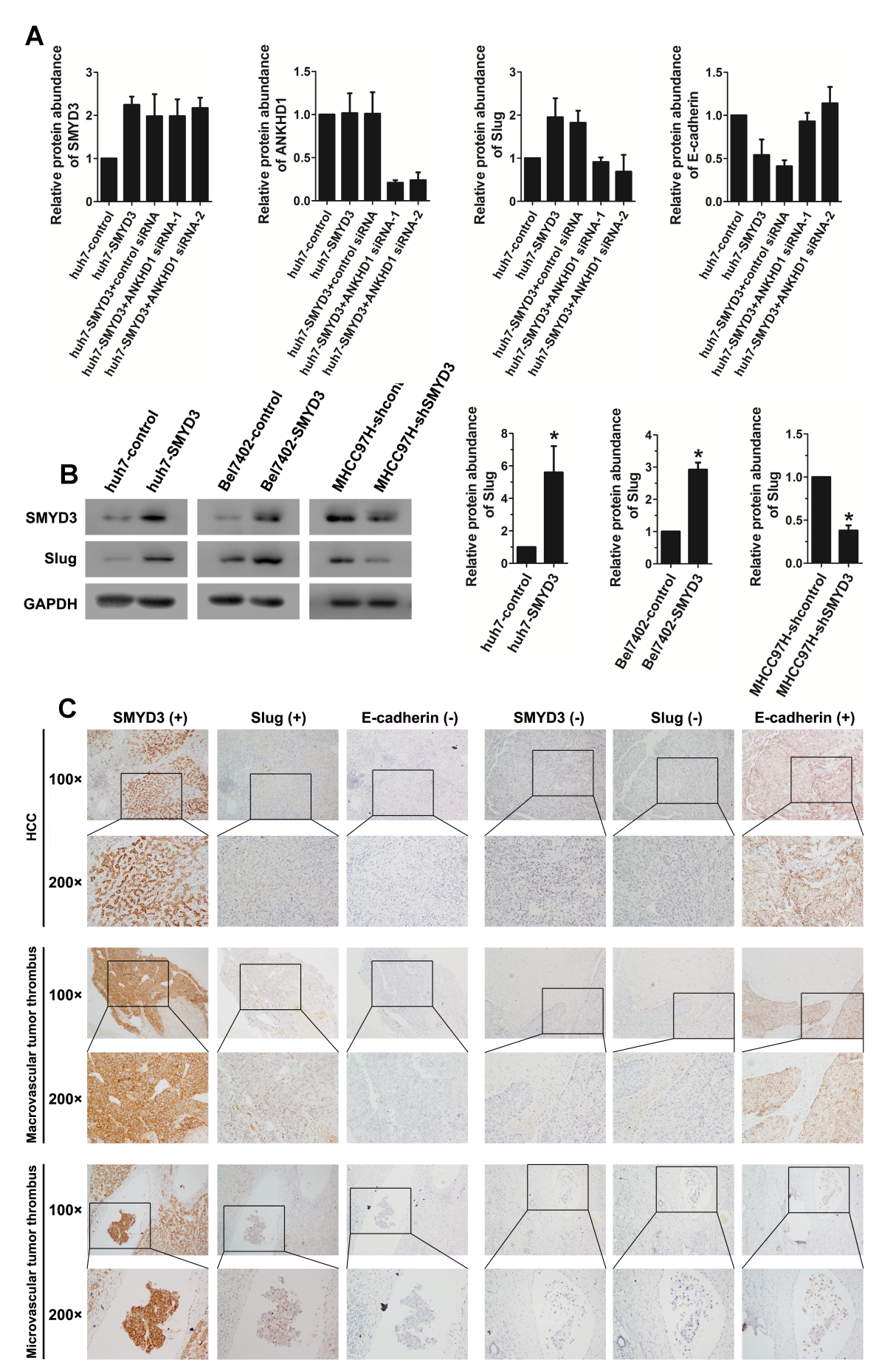


**Figure S5**

**
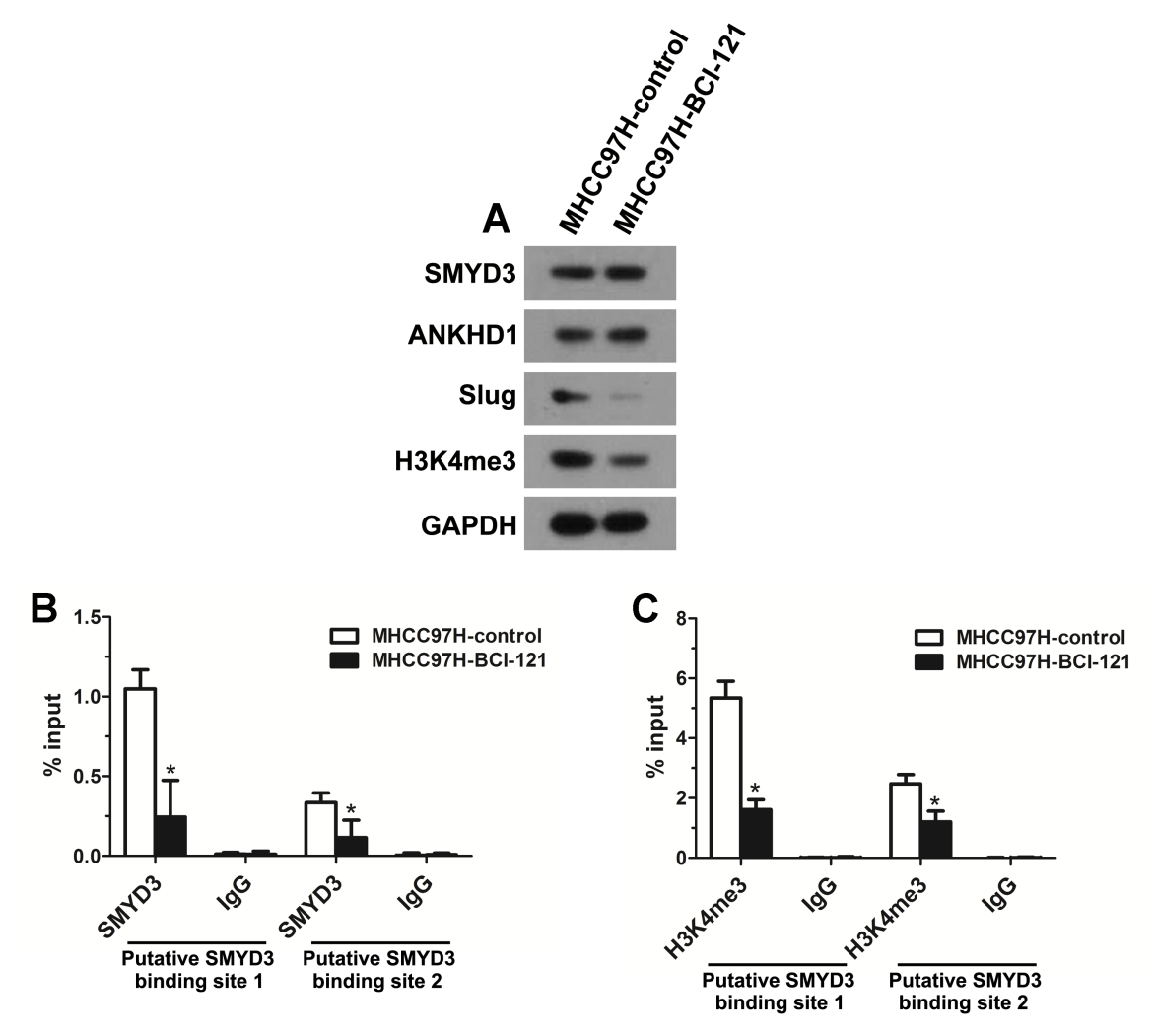
**

**Figure S6**


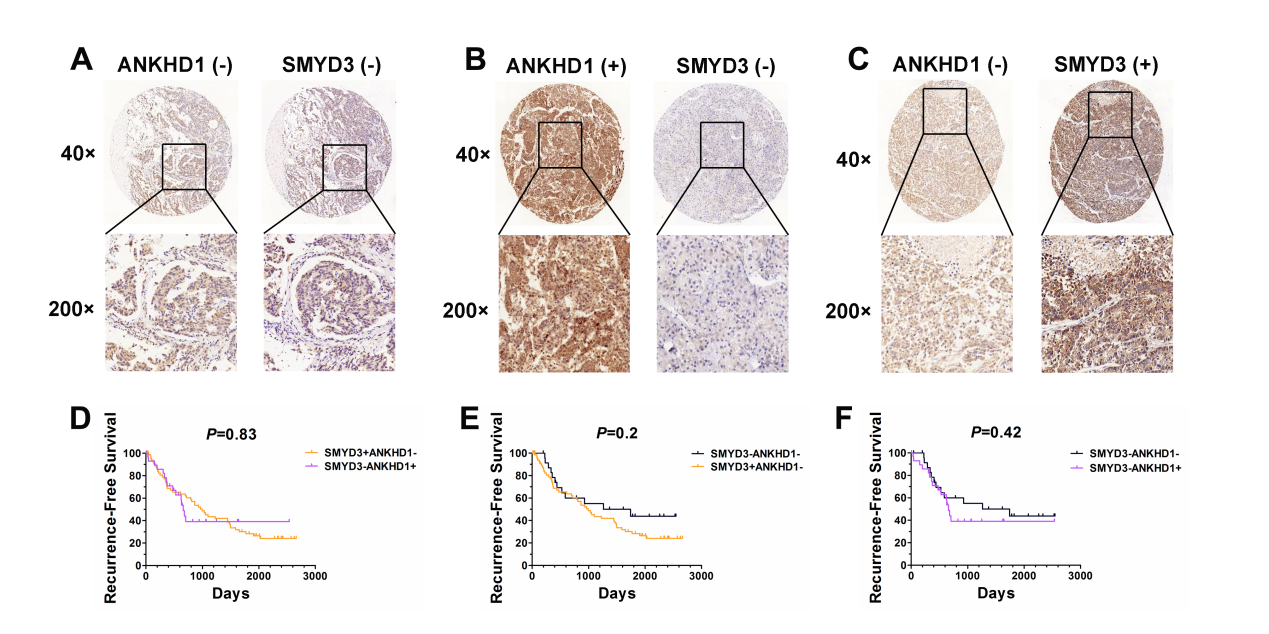


**Figure S7**


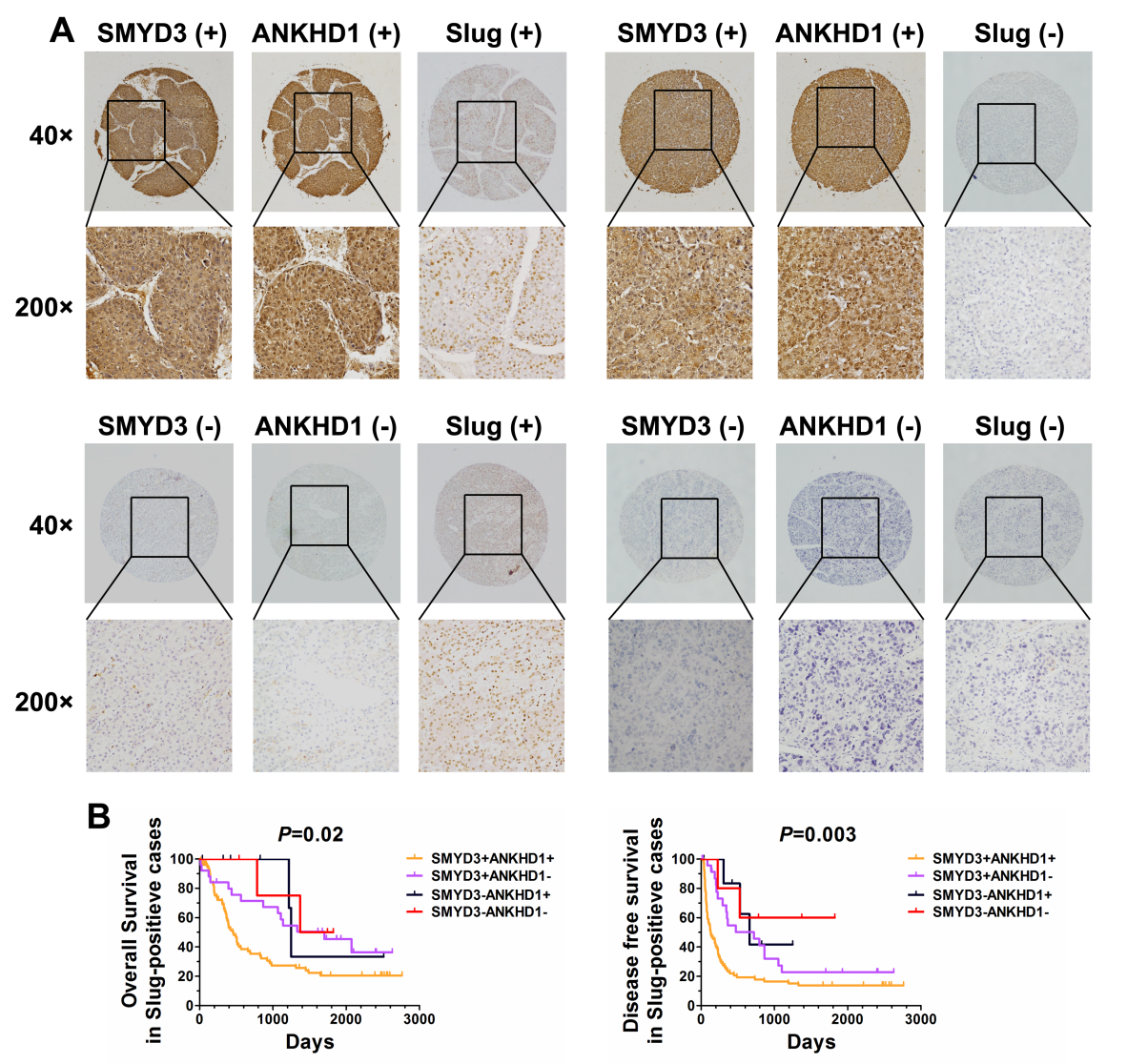

Supplement: Supplementary file 3 — Supplemental figure legends. (DOCX 8542 kb) [file 13046_2018_1011_MOESM3_ESM.docx]
